# 4-(6-Fluoro-1,2-benzisoxazol-3-yl)-1-[2-(2-methyl-4-oxo-6,7,8,9-tetra­hydro-4*H*-pyrido[1,2-*a*]pyrimidin-3-yl)eth­yl]piperidinium nitrate

**DOI:** 10.1107/S1600536809022016

**Published:** 2009-06-20

**Authors:** Yu Sun, Huai-Hong Zhang

**Affiliations:** a Ordered Matter Science Research Center, College of Chemistry and Chemical Engineering, Southeast University, Nanjing 210096, People’s Republic of China; b College of Pharmacy, Jiangsu University, Zhenjiang 212013, People’s Republic of China

## Abstract

In the risperidone cation of the title compound, C_23_H_28_FN_4_O_2_^+^·NO_3_^−^, the piperidine ring adopts a chair conformation and the tetra­hydro­pyridine ring is disordered over two orientations in a 0.620 (11):0.380 (11) ratio. N—H⋯O, C—H⋯O and C—H⋯F hydrogen bonds are present in the crystal structure.

## Related literature

Risperidone is an anti­psychotic agent belonging to a new chemical class of benzisoxazole derivatives, see: Callaghan *et al.* (1999 ▶); Tandon (2002 ▶).
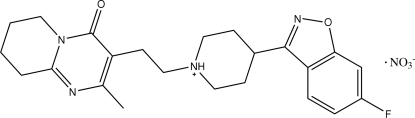


## Experimental

### 

#### Crystal data

C_23_H_28_FN_4_O_2_^+^·NO_3_^−^
*M* *_r_* = 473.50
Monoclinic, 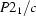

*a* = 8.1655 (16) Å
*b* = 21.866 (4) Å
*c* = 12.635 (3) Å
β = 91.94 (3)°
*V* = 2254.6 (8) Å^3^
*Z* = 4
Mo *K*α radiation
μ = 0.11 mm^−1^
*T* = 293 (2) K
0.15 × 0.12 × 0.09 mm

#### Data collection

Rigaku Scxmini 1K CCD area-detector diffractometer
Absorption correction: multi-scan (*CrystalClear*; Rigaku, 2005) *T*_min_ = 0.94, *T*_max_ = 0.99
22497 measured reflections
5164 independent reflections
3386 reflections with *I* > 2σ(*I*)
*R*_int_ = 0.047

#### Refinement

*R*[*F* ^2^ > 2σ(*F* ^2^)] = 0.067
*wR*(*F* ^2^) = 0.176
*S* = 1.04
5164 reflections
325 parameters
H atoms treated by a mixture of independent and constrained refinement
Δρ_max_ = 0.32 e Å^−3^
Δρ_min_ = −0.28 e Å^−3^

### 

Data collection: *CrystalClear* (Rigaku, 2005 ▶); cell refinement: *CrystalClear*; data reduction: *CrystalClear*; program(s) used to solve structure: *SHELXS97* (Sheldrick, 2008 ▶); program(s) used to refine structure: *SHELXL97* (Sheldrick, 2008 ▶); molecular graphics: *SHELXTL* (Sheldrick, 2008 ▶); software used to prepare material for publication: *SHELXTL*.

## Supplementary Material

Crystal structure: contains datablocks I, global. DOI: 10.1107/S1600536809022016/xu2503sup1.cif

Structure factors: contains datablocks I. DOI: 10.1107/S1600536809022016/xu2503Isup2.hkl

Additional supplementary materials:  crystallographic information; 3D view; checkCIF report

## Figures and Tables

**Table 1:** Hydrogen-bond geometry (Å, °)

*D*—H⋯*A*	*D*—H	H⋯*A*	*D*⋯*A*	*D*—H⋯*A*
N2—H2*B*⋯O3^i^	0.89 (3)	2.07 (3)	2.866 (3)	149 (2)
N2—H2*B*⋯O5^i^	0.89 (3)	2.33 (3)	3.157 (5)	156 (2)
C2—H2*A*⋯O2^ii^	0.93	2.19	3.107 (3)	171
C10—H10*A*⋯O4	0.97	2.50	3.221 (4)	131
C13—H13*B*⋯F^iii^	0.97	2.33	3.264 (3)	161

Symmetry codes: (i) 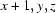; (ii) 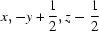; (iii) 
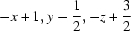
.

**Fig. 1.**
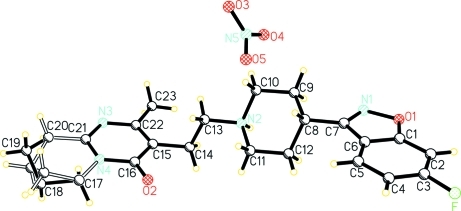
The molecular structure of the title molecule, with the atom-numbering scheme. Displacement ellipsoids are drawn at the 30% probability level.

**Fig. 2.**
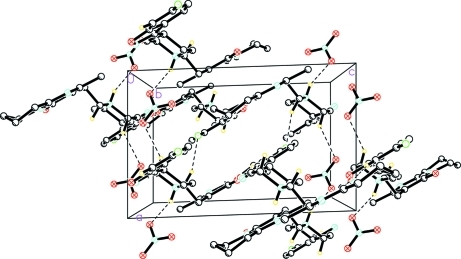
Crystal packing of the compound (I). Hydrogen bonds are shown as dashed lines (Symmetry codes: (i) x, -y + 1/2, z - 1/2; (ii) -x + 1, y - 1/2, -z + 3/2).

## supplementary crystallographic information

### Comment

Risperidone is an antipsychotic agent belonging to a new chemical class of benzisoxazole derivatives, available worldwide since the early 1990s (Callaghan *et al.*, 1999; Tandon, 2002). We report here the synthesis and crystal structure of the title complex, (I) (Fig. 1).

In the structure of (I), there is one risperidone cation and one NO_3_^-^ anion in the asymmetric unit. The expected proton transfer from nitric acid to risperidone occurs at atom N2 of the piperidine ring. Consequently, atom N2 shows quaternary character and bears a positive charge. Compound (I) contains a piperidine ring, one end of which is connected to a pyridopyrimidine group *via* an ethyl bridge, while the other end is connected to an almost-planar fluorobenzisoxazole ring system. In the crystal structure of the title compound, an elaborate hydrogen-bond network is formed. Each NO_3_^-^, one amino group and one carbonyl group in the risperidone molecule are involved in the hydrogen-bond network (Table 1, Fig. 2).

### Experimental

All of the reagents and solvents were purchased from either Aldrich and used without further purification. The compound was recrystallized from a solution in a mixture of methanol and water, with the pH adjusted to 5–6 using 0.1 mol/l HNO_3_, giving colourless crystals of (I) suitable for X-ray diffraction.

### Refinement

The H2B was refined isotropically. H atoms bound to carbon were included in calculated positions and treated in the subsequent refinement as riding atoms, with C—H = 0.96 Å for methyl and 0.97 Å for others, U_iso_(H)=1.2U_eq_(C). The C19 and C20 atoms are disordered over two sites with refined occupancies of 0.620 (11) and 0.380 (11).

### Crystal data

**Table:**

C_23_H_28_FN_4_O_2_^+^·NO_3_^−^	*F*(000) = 1000
*M**_r_* = 473.50	*D*_x_ = 1.395 Mg m^−^^3^
Monoclinic, *P*2_1_/*c*	Mo *K*α radiation, λ = 0.71073 Å
Hall symbol: -P 2ybc	Cell parameters from 4798 reflections
*a* = 8.1655 (16) Å	θ = 3.1–25.0°
*b* = 21.866 (4) Å	µ = 0.11 mm^−^^1^
*c* = 12.635 (3) Å	*T* = 293 K
β = 91.94 (3)°	Block, colourless
*V* = 2254.6 (8) Å^3^	0.15 × 0.12 × 0.09 mm
*Z* = 4	

### Data collection

**Table:**

Rigaku Scxmini 1K CCD area-detector diffractometer	5164 independent reflections
Radiation source: fine-focus sealed tube	3386 reflections with *I* > 2σ(*I*)
graphite	*R*_int_ = 0.047
Detector resolution: 8.192 pixels mm^-1^	θ_max_ = 27.5°, θ_min_ = 3.1°
Thin–slice ω scans	*h* = −10→10
Absorption correction: multi-scan (*CrystalClear*; Rigaku, 2005)	*k* = −28→28
*T*_min_ = 0.94, *T*_max_ = 0.99	*l* = −16→16
22497 measured reflections	

### Refinement

**Table:**

Refinement on *F*^2^	Primary atom site location: structure-invariant direct methods
Least-squares matrix: full	Secondary atom site location: difference Fourier map
*R*[*F*^2^ > 2σ(*F*^2^)] = 0.067	Hydrogen site location: inferred from neighbouring sites
*wR*(*F*^2^) = 0.176	H atoms treated by a mixture of independent and constrained refinement
*S* = 1.04	*w* = 1/[σ^2^(*F*_o_^2^) + (0.07*P*)^2^ + 1.3338*P*] where *P* = (*F*_o_^2^ + 2*F*_c_^2^)/3
5164 reflections	(Δ/σ)_max_ = 0.001
325 parameters	Δρ_max_ = 0.32 e Å^−^^3^
0 restraints	Δρ_min_ = −0.28 e Å^−^^3^

### Special details

**Table:**

Geometry. All esds (except the esd in the dihedral angle between two l.s. planes) are estimated using the full covariance matrix. The cell esds are taken into account individually in the estimation of esds in distances, angles and torsion angles; correlations between esds in cell parameters are only used when they are defined by crystal symmetry. An approximate (isotropic) treatment of cell esds is used for estimating esds involving l.s. planes.
Refinement. Refinement of *F*^2^ against ALL reflections. The weighted *R*-factor *wR* and goodness of fit *S* are based on *F*^2^, conventional *R*-factors *R* are based on *F*, with *F* set to zero for negative *F*^2^. The threshold expression of *F*^2^ > σ(*F*^2^) is used only for calculating *R*-factors(gt) *etc*. and is not relevant to the choice of reflections for refinement. *R*-factors based on *F*^2^ are statistically about twice as large as those based on *F*, and *R*- factors based on ALL data will be even larger.

### Fractional atomic coordinates and isotropic or equivalent isotropic displacement parameters (Å^2^)

**Table:**

	*x*	*y*	*z*	*U*_iso_*/*U*_eq_	Occ. (<1)
F	0.5593 (2)	0.48655 (7)	0.71974 (17)	0.0788 (6)	
C1	0.6901 (3)	0.35617 (11)	0.57834 (19)	0.0394 (6)	
C2	0.6642 (3)	0.41746 (11)	0.5995 (2)	0.0504 (7)	
H2A	0.6986	0.4488	0.5557	0.060*	
C3	0.5836 (3)	0.42758 (11)	0.6905 (2)	0.0511 (7)	
C4	0.5266 (3)	0.38241 (12)	0.7573 (2)	0.0513 (7)	
H4A	0.4691	0.3928	0.8170	0.062*	
C5	0.5565 (3)	0.32220 (11)	0.7336 (2)	0.0438 (6)	
H5A	0.5206	0.2911	0.7773	0.053*	
C6	0.6420 (3)	0.30878 (10)	0.64249 (18)	0.0358 (5)	
C7	0.6994 (3)	0.25504 (10)	0.59070 (17)	0.0353 (5)	
C8	0.6892 (3)	0.19000 (10)	0.62775 (18)	0.0369 (5)	
H8A	0.5777	0.1826	0.6512	0.044*	
C9	0.7256 (3)	0.14414 (11)	0.54190 (19)	0.0454 (6)	
H9A	0.6476	0.1495	0.4830	0.055*	
H9B	0.8343	0.1518	0.5162	0.055*	
C10	0.7164 (3)	0.07913 (11)	0.5818 (2)	0.0477 (6)	
H10A	0.6055	0.0705	0.6026	0.057*	
H10B	0.7427	0.0512	0.5252	0.057*	
C11	0.8011 (4)	0.11368 (11)	0.75999 (19)	0.0488 (7)	
H11A	0.8822	0.1080	0.8170	0.059*	
H11B	0.6940	0.1059	0.7880	0.059*	
C12	0.8082 (4)	0.17918 (11)	0.72137 (19)	0.0470 (6)	
H12A	0.9187	0.1884	0.7005	0.056*	
H12B	0.7816	0.2065	0.7787	0.056*	
C13	0.8196 (3)	0.00410 (10)	0.7117 (2)	0.0403 (6)	
H13A	0.8132	−0.0227	0.6505	0.048*	
H13B	0.7186	−0.0005	0.7491	0.048*	
C14	0.9607 (3)	−0.01635 (11)	0.7837 (2)	0.0427 (6)	
H14A	1.0595	−0.0200	0.7436	0.051*	
H14B	0.9804	0.0135	0.8394	0.051*	
C15	0.9184 (3)	−0.07729 (10)	0.83123 (18)	0.0361 (5)	
C16	0.8370 (3)	−0.07566 (11)	0.9298 (2)	0.0418 (6)	
C21	0.8352 (3)	−0.18542 (10)	0.92195 (18)	0.0370 (5)	
C22	0.9444 (3)	−0.13241 (10)	0.78520 (17)	0.0360 (5)	
C23	1.0216 (3)	−0.13858 (13)	0.6798 (2)	0.0506 (7)	
H23A	1.0477	−0.0987	0.6533	0.076*	
H23B	0.9465	−0.1586	0.6310	0.076*	
H23C	1.1200	−0.1624	0.6876	0.076*	
O1	0.7678 (2)	0.33351 (8)	0.49358 (13)	0.0507 (5)	
O2	0.7981 (3)	−0.02875 (9)	0.97639 (17)	0.0684 (6)	
O3	0.1111 (3)	0.05259 (15)	0.5454 (2)	0.0993 (9)	
O4	0.3344 (3)	0.09708 (14)	0.5171 (2)	0.1005 (9)	
O5	0.1993 (6)	0.11232 (14)	0.6583 (2)	0.1389 (15)	
N1	0.7710 (3)	0.26831 (9)	0.50384 (16)	0.0449 (5)	
N2	0.8321 (2)	0.06899 (8)	0.67408 (14)	0.0329 (4)	
N3	0.9023 (2)	−0.18672 (8)	0.83033 (15)	0.0393 (5)	
N4	0.8013 (2)	−0.13271 (9)	0.97382 (15)	0.0384 (5)	
N5	0.2204 (3)	0.08745 (11)	0.5749 (2)	0.0557 (6)	
C17	0.7250 (4)	−0.13102 (14)	1.0785 (2)	0.0573 (7)	0.620 (11)
H17A	0.6095	−0.1211	1.0697	0.069*	
H17B	0.7771	−0.0998	1.1225	0.069*	
C20	0.7977 (4)	−0.24588 (12)	0.9722 (2)	0.0546 (7)	0.620 (11)
C18	0.7463 (10)	−0.1965 (3)	1.1339 (4)	0.0565 (17)	0.620 (11)
H18A	0.8605	−0.2024	1.1551	0.068*	0.620 (11)
H18B	0.6824	−0.1975	1.1972	0.068*	0.620 (11)
C19	0.6928 (8)	−0.2473 (2)	1.0624 (4)	0.0495 (15)	0.620 (11)
H19A	0.7033	−0.2862	1.0988	0.059*	0.620 (11)
H19B	0.5792	−0.2418	1.0394	0.059*	0.620 (11)
C17'	0.7250 (4)	−0.13102 (14)	1.0785 (2)	0.0573 (7)	0.380 (11)
C20'	0.7977 (4)	−0.24588 (12)	0.9722 (2)	0.0546 (7)	0.380 (11)
H20B	0.8883	−0.2739	0.9632	0.066*	
H20C	0.7004	−0.2635	0.9383	0.066*	
C18'	0.6516 (13)	−0.1831 (4)	1.1149 (6)	0.053 (3)	0.380 (11)
H18C	0.5475	−0.1899	1.0774	0.063*	0.380 (11)
H18D	0.6321	−0.1796	1.1899	0.063*	0.380 (11)
C19'	0.7689 (15)	−0.2360 (4)	1.0947 (8)	0.053 (2)*	0.380 (11)
H19C	0.7247	−0.2732	1.1241	0.064*	0.380 (11)
H19D	0.8733	−0.2278	1.1309	0.064*	0.380 (11)
H2B	0.933 (4)	0.0742 (12)	0.652 (2)	0.052 (8)*	

### Atomic displacement parameters (Å^2^)

**Table:**

	*U*^11^	*U*^22^	*U*^33^	*U*^12^	*U*^13^	*U*^23^
F	0.0694 (11)	0.0375 (9)	0.1310 (17)	0.0082 (8)	0.0251 (11)	−0.0069 (10)
C1	0.0371 (12)	0.0404 (13)	0.0408 (13)	0.0004 (10)	0.0023 (10)	0.0123 (10)
C2	0.0442 (14)	0.0364 (14)	0.0706 (18)	−0.0022 (11)	0.0040 (13)	0.0174 (13)
C3	0.0389 (14)	0.0346 (13)	0.080 (2)	0.0061 (11)	0.0035 (14)	0.0016 (13)
C4	0.0455 (15)	0.0489 (16)	0.0600 (16)	0.0073 (12)	0.0120 (13)	0.0012 (13)
C5	0.0448 (14)	0.0399 (13)	0.0471 (14)	0.0016 (11)	0.0101 (11)	0.0085 (11)
C6	0.0352 (12)	0.0332 (12)	0.0388 (12)	−0.0008 (9)	−0.0006 (10)	0.0076 (9)
C7	0.0370 (12)	0.0379 (12)	0.0307 (11)	0.0013 (10)	0.0001 (10)	0.0045 (9)
C8	0.0397 (12)	0.0337 (12)	0.0378 (12)	−0.0002 (10)	0.0068 (10)	0.0038 (9)
C9	0.0618 (16)	0.0418 (14)	0.0320 (12)	0.0057 (12)	−0.0085 (11)	0.0004 (10)
C10	0.0566 (16)	0.0386 (13)	0.0468 (14)	0.0020 (12)	−0.0156 (12)	−0.0048 (11)
C11	0.0818 (19)	0.0333 (13)	0.0314 (12)	0.0025 (13)	0.0038 (12)	0.0013 (10)
C12	0.0786 (18)	0.0301 (12)	0.0318 (12)	−0.0001 (12)	−0.0053 (12)	0.0015 (9)
C13	0.0447 (13)	0.0273 (11)	0.0490 (14)	−0.0032 (10)	0.0016 (11)	0.0038 (10)
C14	0.0419 (13)	0.0347 (13)	0.0514 (14)	−0.0029 (10)	−0.0015 (11)	0.0070 (11)
C15	0.0369 (12)	0.0330 (12)	0.0383 (12)	0.0000 (9)	0.0005 (10)	0.0036 (9)
C16	0.0482 (14)	0.0325 (12)	0.0448 (14)	0.0031 (11)	0.0025 (11)	−0.0054 (10)
C21	0.0395 (12)	0.0313 (12)	0.0401 (13)	0.0005 (10)	0.0005 (10)	0.0023 (10)
C22	0.0383 (12)	0.0368 (12)	0.0331 (11)	0.0021 (10)	0.0018 (10)	0.0016 (9)
C23	0.0577 (16)	0.0554 (16)	0.0393 (13)	−0.0001 (13)	0.0108 (12)	−0.0011 (12)
O1	0.0646 (12)	0.0439 (10)	0.0444 (10)	−0.0006 (9)	0.0128 (9)	0.0158 (8)
O2	0.0969 (16)	0.0391 (11)	0.0707 (13)	0.0065 (10)	0.0239 (12)	−0.0170 (9)
O3	0.0716 (16)	0.146 (3)	0.0805 (17)	−0.0343 (17)	0.0118 (14)	−0.0153 (17)
O4	0.0505 (13)	0.125 (2)	0.128 (2)	−0.0108 (14)	0.0254 (15)	0.0176 (18)
O5	0.264 (5)	0.081 (2)	0.0734 (18)	−0.020 (2)	0.026 (2)	−0.0229 (16)
N1	0.0574 (13)	0.0401 (11)	0.0376 (11)	0.0001 (10)	0.0068 (10)	0.0080 (9)
N2	0.0332 (10)	0.0319 (10)	0.0337 (10)	−0.0011 (8)	0.0028 (8)	0.0032 (8)
N3	0.0485 (11)	0.0291 (10)	0.0407 (11)	0.0036 (8)	0.0074 (9)	−0.0009 (8)
N4	0.0454 (11)	0.0381 (11)	0.0321 (10)	−0.0019 (9)	0.0048 (8)	−0.0014 (8)
N5	0.0583 (15)	0.0554 (15)	0.0532 (14)	0.0023 (12)	0.0000 (12)	0.0049 (12)
C17	0.0660 (18)	0.072 (2)	0.0350 (13)	−0.0093 (15)	0.0143 (13)	−0.0094 (13)
C20	0.0621 (17)	0.0377 (14)	0.0647 (18)	−0.0015 (12)	0.0104 (14)	0.0125 (12)
C18	0.072 (4)	0.064 (4)	0.033 (2)	−0.007 (3)	0.004 (3)	0.010 (2)
C19	0.052 (3)	0.049 (3)	0.047 (3)	−0.009 (2)	0.003 (2)	0.011 (2)
C17'	0.0660 (18)	0.072 (2)	0.0350 (13)	−0.0093 (15)	0.0143 (13)	−0.0094 (13)
C20'	0.0621 (17)	0.0377 (14)	0.0647 (18)	−0.0015 (12)	0.0104 (14)	0.0125 (12)
C18'	0.051 (6)	0.064 (5)	0.042 (4)	0.004 (4)	0.008 (4)	0.017 (4)

### Geometric parameters (Å, °)

**Table:**

F—C3	1.358 (3)	C14—H14A	0.9700
C1—O1	1.357 (3)	C14—H14B	0.9700
C1—C6	1.381 (3)	C15—C22	1.358 (3)
C1—C2	1.384 (3)	C15—C16	1.432 (3)
C2—C3	1.362 (4)	C16—O2	1.230 (3)
C2—H2A	0.9300	C16—N4	1.400 (3)
C3—C4	1.390 (4)	C21—N3	1.298 (3)
C4—C5	1.374 (4)	C21—N4	1.359 (3)
C4—H4A	0.9300	C21—C20	1.503 (3)
C5—C6	1.398 (3)	C22—N3	1.366 (3)
C5—H5A	0.9300	C22—C23	1.499 (3)
C6—C7	1.431 (3)	C23—H23A	0.9600
C7—N1	1.294 (3)	C23—H23B	0.9600
C7—C8	1.500 (3)	C23—H23C	0.9600
C8—C9	1.514 (3)	O1—N1	1.432 (3)
C8—C12	1.524 (3)	O3—N5	1.222 (3)
C8—H8A	0.9800	O4—N5	1.220 (3)
C9—C10	1.511 (3)	O5—N5	1.204 (3)
C9—H9A	0.9700	N2—H2B	0.89 (3)
C9—H9B	0.9700	N4—C17	1.482 (3)
C10—N2	1.492 (3)	C17—C18	1.600 (7)
C10—H10A	0.9700	C17—H17A	0.9700
C10—H10B	0.9700	C17—H17B	0.9700
C11—N2	1.489 (3)	C20—C19	1.448 (5)
C11—C12	1.515 (3)	C18—C19	1.488 (9)
C11—H11A	0.9700	C18—H18A	0.9700
C11—H11B	0.9700	C18—H18B	0.9700
C12—H12A	0.9700	C19—H19A	0.9700
C12—H12B	0.9700	C19—H19B	0.9700
C13—N2	1.501 (3)	C18'—C19'	1.528 (14)
C13—C14	1.511 (3)	C18'—H18C	0.9700
C13—H13A	0.9700	C18'—H18D	0.9700
C13—H13B	0.9700	C19'—H19C	0.9700
C14—C15	1.507 (3)	C19'—H19D	0.9700
			
O1—C1—C6	109.8 (2)	C13—C14—H14B	109.9
O1—C1—C2	125.8 (2)	H14A—C14—H14B	108.3
C6—C1—C2	124.4 (2)	C22—C15—C16	118.6 (2)
C3—C2—C1	113.7 (2)	C22—C15—C14	124.9 (2)
C3—C2—H2A	123.2	C16—C15—C14	116.4 (2)
C1—C2—H2A	123.2	O2—C16—N4	119.5 (2)
F—C3—C2	117.6 (2)	O2—C16—C15	124.9 (2)
F—C3—C4	117.0 (3)	N4—C16—C15	115.6 (2)
C2—C3—C4	125.4 (2)	N3—C21—N4	123.2 (2)
C5—C4—C3	118.9 (3)	N3—C21—C20	117.1 (2)
C5—C4—H4A	120.6	N4—C21—C20	119.6 (2)
C3—C4—H4A	120.6	C15—C22—N3	123.2 (2)
C4—C5—C6	118.6 (2)	C15—C22—C23	122.5 (2)
C4—C5—H5A	120.7	N3—C22—C23	114.3 (2)
C6—C5—H5A	120.7	C22—C23—H23A	109.5
C1—C6—C5	119.1 (2)	C22—C23—H23B	109.5
C1—C6—C7	104.1 (2)	H23A—C23—H23B	109.5
C5—C6—C7	136.8 (2)	C22—C23—H23C	109.5
N1—C7—C6	111.6 (2)	H23A—C23—H23C	109.5
N1—C7—C8	120.6 (2)	H23B—C23—H23C	109.5
C6—C7—C8	127.8 (2)	C1—O1—N1	107.44 (17)
C7—C8—C9	112.95 (19)	C7—N1—O1	107.01 (19)
C7—C8—C12	110.41 (19)	C11—N2—C10	110.68 (19)
C9—C8—C12	108.51 (19)	C11—N2—C13	111.98 (18)
C7—C8—H8A	108.3	C10—N2—C13	109.88 (18)
C9—C8—H8A	108.3	C11—N2—H2B	108.8 (18)
C12—C8—H8A	108.3	C10—N2—H2B	108.2 (18)
C10—C9—C8	111.8 (2)	C13—N2—H2B	107.1 (18)
C10—C9—H9A	109.3	C21—N3—C22	118.23 (19)
C8—C9—H9A	109.3	C21—N4—C16	121.04 (19)
C10—C9—H9B	109.3	C21—N4—C17	123.4 (2)
C8—C9—H9B	109.3	C16—N4—C17	115.6 (2)
H9A—C9—H9B	107.9	O5—N5—O4	125.4 (3)
N2—C10—C9	111.29 (19)	O5—N5—O3	115.1 (3)
N2—C10—H10A	109.4	O4—N5—O3	119.4 (3)
C9—C10—H10A	109.4	N4—C17—C18	109.0 (3)
N2—C10—H10B	109.4	N4—C17—H17A	109.9
C9—C10—H10B	109.4	C18—C17—H17A	109.9
H10A—C10—H10B	108.0	N4—C17—H17B	109.9
N2—C11—C12	112.12 (19)	C18—C17—H17B	109.9
N2—C11—H11A	109.2	H17A—C17—H17B	108.3
C12—C11—H11A	109.2	C19—C20—C21	119.1 (3)
N2—C11—H11B	109.2	C19—C18—C17	112.1 (5)
C12—C11—H11B	109.2	C19—C18—H18A	109.2
H11A—C11—H11B	107.9	C17—C18—H18A	109.2
C11—C12—C8	111.5 (2)	C19—C18—H18B	109.2
C11—C12—H12A	109.3	C17—C18—H18B	109.2
C8—C12—H12A	109.3	H18A—C18—H18B	107.9
C11—C12—H12B	109.3	C20—C19—C18	107.0 (5)
C8—C12—H12B	109.3	C20—C19—H19A	110.3
H12A—C12—H12B	108.0	C18—C19—H19A	110.3
N2—C13—C14	114.32 (19)	C20—C19—H19B	110.3
N2—C13—H13A	108.7	C18—C19—H19B	110.3
C14—C13—H13A	108.7	H19A—C19—H19B	108.6
N2—C13—H13B	108.7	C19'—C18'—H18C	110.4
C14—C13—H13B	108.7	C19'—C18'—H18D	110.4
H13A—C13—H13B	107.6	H18C—C18'—H18D	108.6
C15—C14—C13	108.73 (19)	C18'—C19'—H19C	109.1
C15—C14—H14A	109.9	C18'—C19'—H19D	109.1
C13—C14—H14A	109.9	H19C—C19'—H19D	107.9
C15—C14—H14B	109.9		
			
O1—C1—C2—C3	−179.6 (2)	C16—C15—C22—N3	−2.6 (4)
C6—C1—C2—C3	−0.7 (4)	C14—C15—C22—N3	−179.2 (2)
C1—C2—C3—F	177.2 (2)	C16—C15—C22—C23	177.2 (2)
C1—C2—C3—C4	−1.5 (4)	C14—C15—C22—C23	0.6 (4)
F—C3—C4—C5	−176.5 (2)	C6—C1—O1—N1	−0.4 (2)
C2—C3—C4—C5	2.2 (4)	C2—C1—O1—N1	178.6 (2)
C3—C4—C5—C6	−0.6 (4)	C6—C7—N1—O1	1.0 (3)
O1—C1—C6—C5	−178.8 (2)	C8—C7—N1—O1	−177.04 (19)
C2—C1—C6—C5	2.2 (4)	C1—O1—N1—C7	−0.4 (2)
O1—C1—C6—C7	0.9 (2)	C12—C11—N2—C10	−55.2 (3)
C2—C1—C6—C7	−178.1 (2)	C12—C11—N2—C13	−178.1 (2)
C4—C5—C6—C1	−1.4 (4)	C9—C10—N2—C11	55.7 (3)
C4—C5—C6—C7	179.0 (3)	C9—C10—N2—C13	179.9 (2)
C1—C6—C7—N1	−1.2 (3)	C14—C13—N2—C11	−72.5 (3)
C5—C6—C7—N1	178.4 (3)	C14—C13—N2—C10	164.0 (2)
C1—C6—C7—C8	176.6 (2)	N4—C21—N3—C22	1.6 (3)
C5—C6—C7—C8	−3.7 (4)	C20—C21—N3—C22	−177.1 (2)
N1—C7—C8—C9	−15.8 (3)	C15—C22—N3—C21	−0.4 (3)
C6—C7—C8—C9	166.5 (2)	C23—C22—N3—C21	179.8 (2)
N1—C7—C8—C12	105.9 (3)	N3—C21—N4—C16	0.4 (3)
C6—C7—C8—C12	−71.8 (3)	C20—C21—N4—C16	179.0 (2)
C7—C8—C9—C10	179.3 (2)	N3—C21—N4—C17	−179.8 (2)
C12—C8—C9—C10	56.6 (3)	C20—C21—N4—C17	−1.2 (3)
C8—C9—C10—N2	−57.8 (3)	O2—C16—N4—C21	176.7 (2)
N2—C11—C12—C8	56.0 (3)	C15—C16—N4—C21	−3.3 (3)
C7—C8—C12—C11	−179.7 (2)	O2—C16—N4—C17	−3.1 (3)
C9—C8—C12—C11	−55.4 (3)	C15—C16—N4—C17	176.9 (2)
N2—C13—C14—C15	169.70 (19)	C21—N4—C17—C18	18.7 (4)
C13—C14—C15—C22	86.2 (3)	C16—N4—C17—C18	−161.5 (3)
C13—C14—C15—C16	−90.5 (3)	N3—C21—C20—C19	−167.1 (4)
C22—C15—C16—O2	−175.7 (2)	N4—C21—C20—C19	14.2 (5)
C14—C15—C16—O2	1.1 (4)	N4—C17—C18—C19	−50.2 (7)
C22—C15—C16—N4	4.3 (3)	C21—C20—C19—C18	−43.9 (7)
C14—C15—C16—N4	−178.9 (2)	C17—C18—C19—C20	62.0 (7)

### Hydrogen-bond geometry (Å, °)

**Table:**

*D*—H···*A*	*D*—H	H···*A*	*D*···*A*	*D*—H···*A*
N2—H2B···O3^i^	0.89 (3)	2.07 (3)	2.866 (3)	149 (2)
N2—H2B···O5^i^	0.89 (3)	2.33 (3)	3.157 (5)	156 (2)
C2—H2A···O2^ii^	0.93	2.19	3.107 (3)	171
C10—H10A···O4	0.97	2.50	3.221 (4)	131
C13—H13B···F^iii^	0.97	2.33	3.264 (3)	161

Symmetry codes: (i) *x*+1, *y*, *z*; (ii) *x*, −*y*+1/2, *z*−1/2; (iii) −*x*+1, *y*−1/2, −*z*+3/2.
